# Sleep patterns and quality among Nigerian medical students: A cross-sectional study

**DOI:** 10.1097/MD.0000000000037556

**Published:** 2024-03-22

**Authors:** Nicholas Aderinto, Gbolahan Olatunji, Samson Afolabi, Abdulbasit Muili, Peter Olaniyi, Mariam Edun, Damilare Olakanmi

**Affiliations:** a Department of Medicine and Surgery, Ladoke Akintola University of Technology, Ogbomoso, Nigeria; b Department of Medicine and Surgery, University of Ilorin, Ilorin, Nigeria.

**Keywords:** academic performance, medical students, Nigeria, sleep patterns, sleep quality

## Abstract

Adequate sleep is crucial for individuals’ well-being and cognitive functioning. However, medical students face unique challenges that disrupt their sleep patterns, such as a rigorous curriculum, long study hours, and high-stress levels. Understanding the sleep patterns and quality among medical students in Nigeria is important to develop targeted interventions and support their overall well-being. This study involved 802 medical students from 3 medical schools in Southwest Nigeria. Participants completed an online questionnaire that collected data on their demographic characteristics, sleep patterns and self-reported sleep quality. Descriptive statistics and correlation analysis were used to analyze the data and identify patterns and associations. Most participants were female (56.9%), with the highest representation from the UNILORIN (65.5%). The average reported sleep duration was 5.74 hours per night, indicating insufficient sleep. Irregular bedtimes and wake-up times were commonly reported. A significant proportion of students consumed coffee late at night (27.1%) and used medication to induce sleep (24.3%). Sleep patterns and behaviors, such as snoring (36.1%) and nocturnal eating (57.6%), were reported. Overall, participants reported satisfactory (28.3%) or poor (29.7%) sleep quality. Correlation analysis revealed significant associations between sleep patterns, sleep quality, academic performance, and other sleep-related factors. The study identified insufficient sleep duration, irregular bedtimes, late-night coffee consumption, and poor sleep quality. These findings emphasize the need for interventions and strategies to promote healthy sleep habits among medical students, which can positively impact their overall health and academic performance.

## 1. Introduction

Sleep is a fundamental physiological activity critical in cognitive processes, memory consolidation, and overall well-being.^[1,2]^ However, poor sleeping habits are commonly observed among students, including medical students, due to the demanding nature of their academic pursuits.^[3]^ Insufficient sleep duration and irregular sleep schedules are often reported, particularly during exam periods when students resort to staying late to study.^[3]^ Consequently, these sleep disturbances can lead to excessive daytime sleepiness, reduced attentiveness during lectures, and disruptions in daily functioning. They may even contribute to developing insomnia and mental health disorders.^[3]^

Although this study is the first to assess sleep patterns and quality, specifically among medical students in Nigeria, similar studies conducted in other countries have shown comparable results. For example, Lawson et al found poor sleep quality in 56.2% of Ghanaian students, associated with morning tiredness, sleepiness during lectures, poor academic performance, and insomnia-related conditions.^[4]^ Additionally, research has indicated that sleep disorders are influenced by various factors such as age, gender, and lifestyle. Age is recognized as a significant determinant of sleep patterns, with an increased risk of sleep disorders observed as individuals age.^[4]^ Moreover, female students are more susceptible to sleep disorders than their male counterparts. Lifestyle factors, including dietary habits, smoking, excessive coffee consumption, and exercise, have also been linked to sleep disorders among medical students.^[4–6]^

The sleep quality of medical students is not merely a personal concern; it directly relates to their academic performance and overall educational experience.^[5]^ Sleep deprivation has been linked to decreased cognitive function, impaired memory, and reduced learning efficiency.^[7,8]^ Moreover, inadequate sleep is associated with a range of health issues, including increased stress levels, compromised immune function, and a higher risk of mental health disorders.^[9]^ Given the demanding nature of medical education, understanding the health implications of poor sleep is crucial for implementing targeted interventions to promote the well-being of medical students. The sleep patterns established during medical education can affect physicians’ professional lives.^[10]^ Sleep-deprived individuals may face challenges in decision-making, patient interactions, and overall job performance.^[11]^

Recognising and addressing sleep-related challenges could positively impact individual well-being and academic success, improving the overall quality of medical education. As institutions worldwide increasingly recognize the importance of student well-being, the outcomes of this study may provide valuable input for shaping policies and practices within Nigerian medical schools.^[12]^ Implementing evidence-based strategies to support sleep health could contribute to a more sustainable and supportive learning environment. This current study aims to assess sleeping patterns and quality among medical students in Nigeria.

## 2. Methodology

### 2.1. Study design

This study followed a cross-sectional design to investigate sleep patterns and quality among Nigerian medical students.

### 2.2. Study setting

The study comprised medical students pursuing their undergraduate degrees at University of Ilorin, Ladoke Akintola University of Technology (LAUTECH) and Osun State University (UNIOSUN). These universities were selected as study sites on their reputations for providing high-quality undergraduate medical education in Nigeria. The universities attract students from diverse regions of Nigeria and beyond and have modern facilities and teaching resources that support research activities.

### 2.3. Participants

The participants consisted of medical students enrolled in Nigerian medical schools. A convenience sampling method was utilized to recruit participants from multiple medical schools across different regions of Nigeria. Inclusion criteria included being a current medical student and providing informed consent to participate in the study.

### 2.4. Data collection

Data collection involved utilizing a questionnaire consisting of 39 items. The questionnaire was adapted from a previous study by Sweileh et al in 2011.^[5]^ It was designed based on the Diagnostic and Statistical Manual of Mental Disorders IV criteria for sleep disorders and the Pittsburgh Sleep Quality Index (PSQI). The questionnaire used in this study consisted of 6 sections that covered various domains. The demographic characteristics section included items related to gender, age, program, entry type, academic level, and hostel of residence. The sleep habits section assessed variables such as the time of going to bed, hours of sleep, time of waking, nocturnal intake of coffee, and use of sleeping pills. The sleep problems section included the time to fall asleep, the number of times one wakes up during the night, snoring, and reasons for failing to maintain sleep. The parasomnia section explored sleep-talking, sleep-walking, and nightmares. The daytime tiredness and sleepiness section assessed feelings of tiredness in the morning, daytime sleepiness during lectures and free time, and daytime naps. The general subjective questions section covered feelings about sleep quality, sleep quality on the night before an exam, academic achievement, feelings about leisure time, and living conditions. The PSQI questionnaire, which comprised 19 self-rated questions that generated 7 composite scores, was used. These scores were categorized into subjective sleep quality, sleep latency, sleep duration, habitual sleep efficiency, sleep disturbances, use of sleep medication, and daytime dysfunction. The PSQI questionnaire has demonstrated good psychometric properties and has been validated among various student populations worldwide, including West Africa.^[13]^ Consistent with other populations, a global sum score greater than 5 indicated poor sleep quality. A pilot study was conducted before data collection to enhance the questionnaire’s validity and reliability. A small sample of medical students participated in the pilot study and provided feedback on the questionnaire items’ clarity, comprehensibility, and relevance.

### 2.5. Ethical considerations

Ethical approval for this study was obtained from the Research Ethics Committee of each participating medical school. Participants were fully informed about the study’s purpose, procedures, and rights, including voluntary participation and the ability to withdraw at any time. Confidentiality and anonymity were assured, and no personally identifiable information was collected. Informed consent was obtained from all participants before they participated in the study.

### 2.6. Data analysis

The collected data were analyzed using SPSS 25.0. Descriptive statistics were employed to summarize the demographic characteristics of the participants, as well as the various sleep-related variables assessed in the study. Continuous variables like sleep duration were presented as means and standard deviations, while categorical variables were reported as frequencies and percentages. Inferential statistical analyses explored potential associations between sleep patterns and demographic variables factors. Correlation analyses, such as Pearson’s correlation coefficient, were utilized to examine relationships between continuous variables like sleep duration and academic level.

## 3. Result

Table 1 summarizes the essential demographic characteristics of the study population. Among the participants, 456 were females (56.9%), and 346 were males (43.1%). The age distribution revealed that 165 participants (20.6%) were under 20 years old, while the majority, 616 participants (76.8%), fell between 20 and 25. Only 21 participants (2.6%) were above 25 years of age. Regarding the educational institutions attended, 180 individuals (22.4%) were from LAUTECH, 525 (65.5%) were from UNILORIN, and 97 (12.1%) were from UNIOSUN. The academic level of the participants varied across different stages, with 27 individuals (3.4%) in the 100 level, 201 (25.1%) in the 200 level, 231 (28.8%) in the 300 level, 173 (21.6%) in the 400 level, 104 (13.0%) in the 500 level, and 66 (8.2%) in the 600 level.

**Table 1:** Demographic characteristics of the study population.

		Freq	Percentage
Gender	Female	456	56.9
	Male	346	43.1
Age (yr)	<20	165	20.6
	20–25	616	76.8
	>25	21	2.6
School	LAUTECH	180	22.4
	UNILORIN	525	65.5
	UNIOSUN	97	12.1
Academic level	100	27	3.4
	200	201	25.1
	300	231	28.8
	400	173	21.6
	500	104	13.0
	600	66	8.2

Table 2 displays the sleep characteristics of the study participants. Participants reported an average work duration of 5.09 ± 3.441 hours during the day and 3.86 ± 1.63 hours at night. The participants’ average sleep duration at night was 5.74 ± 0.995 hours. Regarding waking up in the morning, the most common preference was to wake up between 6 am and 8 am, accounting for 62% of respondents. Bedtime preferences were also diverse, with 54.6% of participants sleeping between 10 pm and 12 am. Regarding nighttime habits, most participants (72.8%) reported never drinking coffee late at night, 17.6% used sleeping pills occasionally, and 6.7% reported regular usage. Regarding the time it takes to fall asleep, nearly 40% of participants mentioned taking 10-30 minutes, while 63.7% reported never snoring.

**Table 2:** Sleep characteristics of participants.

		Freq	Percentage
Hours of work during the day (N = 291)	5.09 ± 3.441		
Hours of work at night (N = 94)	3.86 ± 1.63		
Sleep duration at night (Mean ± SD)	5.74 ± 0.995		
Wakeup times	1.65 ± 0.884		
Go to sleep	<10 pm	113	14.1
	>12 am	233	29.1
	10 pm–12 am	438	54.6
	No fixed time	18	2.2
Usually, Wake up	<12 am	2	0.2
	12–4 am	13	1.6
	4–6 am	172	21.4
	6–8 am	497	62
	8 am–12 pm	30	3.7
	Around 12 pm–2 pm	82	10.2
	Depends	6	0.7
Drink coffee late in the night	Never	584	72.8
	Less than once a week	208	25.9
	1–2 nights a week	4	0.5
	3–4 nights a week	5	0.6
	Nightly/daily	1	0.1
Use sleeping pills to induce sleep	No	607	75.7
	Sometimes	141	17.6
	Yes	54	6.7
The time it takes to fall asleep	<10 min	237	29.6
	10–30 min	311	38.8
	30–60 min	170	21.2
	>60 min	84	10.5
Snore	Never	511	63.7
	Less than once a week	269	33.5
	1–2 nights a week	14	1.7
	3–4 nights a week	2	0.2
	Nightly/daily	6	0.7

**Figure 1.**
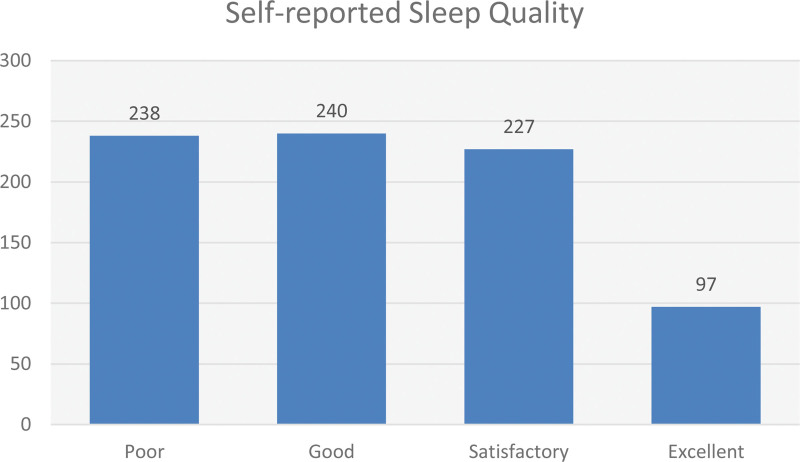
Self-reported sleep quality.

Regarding self-reported sleep quality, the responses from the participants indicated varying levels of sleep quality. Out of the total respondents, 97 individuals (12.1%) reported experiencing excellent sleep quality, while 240 participants (29.9%) reported good sleep quality. Additionally, 227 respondents (28.3%) reported having satisfactory sleep quality, while 238 participants (29.7%) reported poor sleep quality (Fig. 1).

Table 3 provides a comprehensive overview of the sleep patterns and frequency among the study participants, shedding light on various sleep-related behaviors and experiences. Waking up due to noise was reported by varying percentages of participants. Notably, 21.4% reported never experiencing this, while 24.2% mentioned it occurring less than once a week. For 16.7%, it happened 1 to 2 nights a week, and 19.1% experienced it 3 to 4 nights a week. A further 18.6% reported encountering this issue nightly or daily.

**Table 3:** Sleep patterns and frequency of occurrence.

	Never	Less than once a week	1–2 nights a week	3–4 nights a week	Nightly/daily
Wake up due to noise	172 (21.4%)	194 (24.2%)	134 (16.7%)	153 (19.1%)	149 (18.6%)
Nocturnal eating	462 (57.6%)	324 (40.4%)	14 (1.7%)	1 (0.1%)	1 (0.1%)
Sleepwalking	791 (98.6%)	9 (1.1%)	2 (0.2%)	(0%)	(0%)
Sleep talking	505 (63%)	182 (22.7%)	100 (12.5%)	4 (0.5%)	11 (1.4%)
Nocturnal bruxism	784 (97.8%)	8 (1%)	9 (1.1%)	1 (0.1%)	(0%)
Restless leg syndrome	359 (44.8%)	21 (2.6%)	34 (4.2%)	233 (29.1%)	155 (19.3%)
Nightmares	315 (39.3%)	213 (26.6%)	264 (32.9%)	7 (0.9%)	3 (0.4%)
Feeling tired in the morning	120 (15%)	88 (11%)	200 (24.9%)	288 (35.9%)	106 (13.2%)
Daytime sleepiness	63 (7.9%)	190 (23.7%)	383 (47.8%)	145 (18.1%)	21 (2.6%)
Daytime sleepiness during lectures	89 (11.1%)	124 (15.5%)	350 (43.6%)	196 (24.4%)	43 (5.4%)
Daytime sleepiness during free times	171 (21.3%)	58 (7.2%)	246 (30.7%)	236 (29.4%)	91 (11.3%)
Daytime nap	168 (20.9%)	369 (46%)	108 (13.5%)	136 (17%)	21 (2.6%)

Nocturnal eating behaviors were observed in the study population, with 57.6% indicating that they never engaged in this behavior. However, 40.4% reported doing it less than once a week, and only a small percentage engaged more frequently. Sleepwalking was rare, with the majority (98.6%) reporting never experiencing it. A small fraction (1.1%) reported it happening less than once a week.

Sleep talking was reported by 63% of participants, with varying frequencies. Some experienced it less than once a week (22.7%), while others reported it occurring 1 to 2 nights a week (12.5%) or even more frequently. Nocturnal bruxism (teeth grinding) was rare, with 97.8% of participants never experiencing it. A small number (1.1%) reported it happening 1 to 2 nights a week. Restless leg syndrome affected 44.8% of participants, occurring less than once a week. However, a significant portion experienced it more frequently, with 29.1% reporting 3 to 4 nights a week and 19.3% experiencing it nightly or daily.

Nightmares were reported by 39.3% of participants, again with varying frequencies. Some experienced them less than once a week (26.6%), while others reported more frequent nightmares. Feeling tired in the morning was reported by 15% of participants who never experienced it. However, a substantial number (35.9%) reported feeling tired 3 to 4 nights a week, with 13.2% feeling tired nightly or daily. Daytime sleepiness was observed in varying degrees, with 7.9% never feeling sleepy during the day. Some participants reported feeling sleepy less than once a week (23.7%), while others experienced it more frequently. During lectures, 11.1% of participants never experienced daytime sleepiness. However, 43.6% reported feeling sleepy 1 to 2 nights a week during lectures, with varying frequencies for others. During free time, 21.3% of participants never experienced daytime sleepiness. Like lectures, daytime sleepiness during free time varied in frequency among participants. Daytime napping behavior was diverse, with 20.9% reporting never taking daytime naps. The frequency of napping ranged from less than once a week to nightly or daily for different participants.

Tables 4 and 5 display the correlations between various sleep patterns and sleep quality among the study population. The table provides insights into the relationships between variables and overall sleep quality. Academic level negatively correlated with sleep quality, with a correlation coefficient of −0.100 (*P* = .004*). Work during the day positively correlated with sleep quality, with a correlation coefficient of 0.102 (*P* = .004*). Work at night showed a negative correlation with sleep quality, with a correlation coefficient of −0.122 (*P* = .001*). Time taken to fall asleep (sleep latency) showed a positive correlation with sleep quality, with a coefficient of 0.412 (*P* = .000*). Coffee consumption late at night showed a strong positive correlation with sleep quality, with a correlation coefficient of 0.386 (*P* = .000*). The use of sleeping pills showed a strong positive correlation with sleep quality, with a correlation coefficient of 0.397 (*P* = .000*). Time of sleep showed a positive correlation with sleep quality, with a correlation coefficient of 0.368 (*P* = .000*). Sleep hours positively correlated with sleep quality, with a correlation coefficient of 0.072 (*P* = .041*). Wake-up time positively correlated with sleep quality, with a correlation coefficient of 0.111 (*P* = .002*). Sleep talking positively correlated with sleep quality, with a correlation coefficient of 0.086 (*P* = .015*). Restless leg syndrome showed a strong positive correlation with sleep quality, with a correlation coefficient of 0.420 (*P* = .000*). Nightmares showed a strong positive correlation with sleep quality, with a correlation coefficient of 0.569 (*P* = .000*). Feeling tired in the morning showed a strong positive correlation with sleep quality, with a correlation coefficient of 0.616 (*P* = .000*). Daytime sleepiness showed a strong positive correlation with sleep quality, with a correlation coefficient of 0.420 (*P* = .000*). Daytime sleepiness during lectures showed a strong positive correlation with sleep quality, with a correlation coefficient of 0.450 (*P* = .000*). Daytime sleepiness during free times positively correlated with sleep quality, with a correlation coefficient of 0.317 (*P* = .000*). Daytime nap showed a positive correlation with sleep quality, with a correlation coefficient of 0.257 (*P* = .000*). Sleep quality on the night before exams showed a strong positive correlation, with a correlation coefficient of 0.510 (*P* = .000*). Leisure time showed a strong positive correlation with sleep quality, with a correlation coefficient of 0.536 (*P* = .000*). Living conditions positively correlated with sleep quality, with a correlation coefficient of 0.413 (*P* = .000*).

**Table 4:** Correlation between participants’ variables and sleep quality.

Variable	Sleep quality	
	Correlation coefficient	*P* value
Academic level	−0.100	.004
Work during the day	0.102	.004
Work at Night	−0.122	.001
Time sleep	0.368	.000
Sleep hours	0.072	.041
Time wakeup	0.111	.002
Take coffee late in the night	0.386	.000
Use sleep pills	0.397	.000
Sleep latency	0.412	.000
Wake up times during sleep	0.006	.884
Snore	0.018	.614
Wake up due to noice	0.314	.000
Nocturnal eating	−0.072	.041
Sleepwalking	−0.016	.660
Sleep talking	0.086	.015
Nocturnal bruxism	−0.067	.056
Restless leg syndrome	0.420	.000
Nightmares	0.569	.000
Feeling tired in the morning	0.616	.000
Daytime sleepiness	0.420	.000
Daytime sleepiness during lectures	0.450	.000
Daytime sleepiness during free times	0.317	.000
Daytime nap	0.257	.000
Sleep quality on night before exams	0.510	.000
Leisure time	0.536	.000
Living condition	0.413	.000

**Table 5:** Correlation between participants’ variables and sleep quality.

Variable	Coefficients		*t* statistic	*P* value
	B	Std Error		
Academic level	−0.001	0.00	−3.332	.001
Work during the day	0.030	0.010	3.112	.002
Work at night	−0.058	0.019	−2.981	.003
Time sleep	0.275	0.049	5.643	.000
Sleep hours	−0.136	0.027	−4.992	.000
Time wakeup	−0.016	0.035	−0.446	.655
Take coffee late in the night	0.064	0.047	1.357	.175
Use sleep pills	0.188	0.062	3.033	.003
Sleep latency	0.072	0.042	1.714	.087
Wake up times during sleep	−0.120	0.032	−3.707	.000
Snore	0.013	0.018	0.721	.471
Wake up due to noise	0.194	0.026	7.491	.000
Nocturnal eating	−0.037	0.042	−0.883	.377
Sleepwalking	−0.073	0.111	−0.663	.508
Sleep talking	−0.027	0.026	−1.021	.308
Nocturnal bruxism	−0.187	0.064	−2.923	.004
Restless leg syndrome	0.228	0.027	8.430	.000
Nightmares	0.079	0.031	2.550	.011
Feeling tired in the morning	0.185	0.027	6.834	.000
Daytime sleepiness	−0.077	0.037	−2.076	.038
Daytime sleepiness during lectures	−0.074	0.030	−2.458	.014
Daytime sleepiness during free times	0.108	0.033	3.327	.001
Daytime nap	0.116	0.033	3.536	.000
Sleep quality on night before exams	0.152	0.038	4.026	.000
Leisure time	0.272	0.036	7.563	.000
Living condition	0.055	0.043	0.576	.565
Model summary				
*R* square	0.875			
Adjusted *R* square	0.869			
SE. of regression	0.405			

Table 6 summarizes the connection between academic performance and sleep quality within our study’s participant pool. In the category of excellent academic performance, there were 346 participants, constituting 43.1% of the total. Among them, 23.4% reported excellent sleep quality, 48.8% reported good sleep quality, 2.3% reported satisfactory sleep quality, and 25.4% reported poor sleep quality. For those with good academic performance, totaling 162 participants (20.2%), 4.3% experienced excellent sleep quality, 35.2% reported good sleep quality, 58% had satisfactory sleep quality, and 2.5% reported poor sleep quality. Participants with satisfactory academic performance numbered 291 (36.3%). Of them, 3.1% had excellent sleep quality, 4.5% reported good sleep quality, 42.6% had satisfactory sleep quality, and 49.8% experienced poor sleep quality. In the poor academic performance category, there were only 3 participants (0.4%), and they all reported poor sleep quality. One-third reported good, satisfactory, and poor sleep quality.

**Table 6:** Academic performance and sleep quality.

Academic performance	Sleep quality n (%)				
	Excellent	Good	Satisfactory	Poor	Total
Excellent	81 (23.4)	169 (48.8)	8 (2.3)	88 (25.4)	346 (43.1)
Good	7 (4.3)	57 (35.2)	94 (58)	4 (2.5)	162 (20.2)
Satisfactory	9 (3.1)	13 (4.5)	124 (42.6)	145 (49.8)	291 (36.3)
Poor	0 (0)	1 (33.3)	1 (33.3)	1 (33.3)	3 (0.4)
Total	97 (12.1)	240 (29.9)	227 (28.3)	238 (29.7)	802 (100)

Chi-square = 407.058, df = 9, *P* = .000

* [

* *P* value < .05].

## 4. Discussion

The demographic distribution of participants in our cross-sectional study on sleep patterns and quality among Nigerian medical students revealed a higher representation of female students than male students, consistent with the global trend of increasing female enrollment in medical education.^[14]^ This observation highlights the importance of considering gender-related factors when understanding sleep patterns in this population. It is worth noting that gender distributions among medical students have shown variations in different geographic contexts, as observed in studies conducted in Palestine and Brazil.^[15,16]^ These variations in gender representation within the medical student population may provide valuable insights into potential differences in sleep patterns.^[17]^ For instance, Alsaggaf et al reported a higher prevalence of sleep disorders among female students.^[18]^ Therefore, our study’s findings on gender distribution underscore the significance of considering gender-related factors when examining sleep patterns among Nigerian medical students.^[18]^

Regarding age distribution, most participants in our study fell within the typical undergraduate age range of 20-25 years, consistent with previous research findings.^[5,8,19]^ This age range corresponds to the phase of medical education and may have implications for sleep patterns, as sleep disorders tend to be more prevalent among older individuals.^[20]^ Consequently, the demographic findings in our study provide valuable contextual information for interpreting and generalizing the study’s findings on sleep patterns and disorders among medical students. Furthermore, they emphasize the importance of considering age-related factors when examining this population’s prevalence and potential variations in sleep patterns.

Our study revealed an average sleep duration at night of 5.74 ± 0.995 hours among Nigerian medical students, indicating insufficient sleep and aligning with previous studies conducted in similar populations. This finding highlights the challenge medical students face in attaining adequate sleep duration, which can affect cognitive function and pose health risks associated with sleep deprivation.^[21]^ Thus, addressing this issue within Nigerian medical education is crucial. Additionally, our study identified variations in participants’ bedtime and waking time preferences, influenced by personal routines, academic demands, and lifestyle factors. These findings underscore the importance of tailoring sleep management interventions to accommodate the specific needs of Nigerian medical students to optimize sleep schedules and promote improved sleep quality and overall well-being.

Most participants in our study reported abstaining from coffee late at night, aligning with recommendations to limit caffeine intake close to bedtime.^[22,23]^ This awareness highlights the importance of promoting sleep hygiene practices and educating students about the potential effects of caffeine on sleep. Medical students can enhance their sleep quality and well-being by addressing factors that disrupt sleep, such as late-night caffeine consumption. Similarly, a relatively lower prevalence of sleep aid usage was observed among Nigerian medical students compared to other populations. This suggests the need to explore non-pharmacological interventions and address underlying sleep difficulties. Strategies such as cognitive-behavioral therapy for insomnia, relaxation techniques, and sleep hygiene education can provide effective alternatives to promote healthy sleep practices among Nigerian medical students.

Our study found variations in the time to fall asleep, with a significant proportion of participants experiencing quick sleep onset. This finding is consistent with previous studies among medical students^[24,25]^ and emphasizes the importance of considering stress levels, pre-sleep routines, and environmental conditions in optimizing sleep initiation. Interventions targeting these factors can improve sleep initiation and promote faster sleep onset among Nigerian medical students. Additionally, the prevalence of snoring among Nigerian medical students was notable, with the majority reporting never snoring and a significant proportion snoring less than once a week. These findings align with previous studies among medical students^[15,26]^ and highlight the importance of considering potential sleep-disordered breathing issues, such as obstructive sleep apnea, for improving sleep quality and overall well-being within this population.

In terms of self-reported sleep quality, our study revealed a range of experiences among Nigerian medical students, with participants reporting varying levels of sleep quality. Notably, a subset of individuals reported excellent (12.1%) and good (29.9%) sleep quality, suggesting favorable sleep outcomes within the study population. These findings are consistent with previous research conducted among similar populations, highlighting the prevalence of suboptimal sleep quality among medical students.^[16,18,27]^ While some participants reported satisfactory and poor sleep quality, which raises concerns due to the negative outcomes associated with such sleep disturbances, understanding the underlying factors contributing to suboptimal sleep quality in this population is essential for developing effective interventions. The demanding nature of medical education, including long study hours, clinical rotations, and high-stress levels, has been consistently identified as a potential contributor to poor sleep quality among medical students.^[5,7]^

Additionally, lifestyle factors such as irregular sleep schedules, excessive use of electronic devices, and inadequate physical activity may play a role.^[10]^ Future research should explore the specific determinants of sleep quality among Nigerian medical students, considering the unique contextual factors in this population. Longitudinal studies could provide insights into the trajectory of sleep quality throughout medical education and its potential impact on future professional practice. Moreover, interventions targeting modifiable factors, such as workload management and stress reduction techniques, should be developed and evaluated for their effectiveness in improving sleep quality and overall well-being.

Our study also investigated various sleep-related behaviors among Nigerian medical students. Waking up due to noise was reported by varying proportions of participants, suggesting the need to address noise-related sleep disruptions and create conducive sleeping environments for this population. While nocturnal eating behaviors were relatively infrequent, addressing them is still important to promote optimal sleep quality and overall health. Sleepwalking was rare among participants, aligning with the low prevalence reported in the literature.^[28]^ However, individuals who experience sleepwalking may benefit from interventions to manage and reduce sleepwalking episodes.^[26]^ Sleep talking, reported by many participants, may indicate other sleep-related issues and should be further explored. A small percentage of participants reported nocturnal bruxism (teeth grinding), suggesting the need for further evaluation and management in those affected. Restless leg syndrome was prevalent among Nigerian medical students, underscoring the importance of identifying and addressing this sleep disorder. The considerable proportion of participants reporting nightmares, feelings of tiredness in the morning, and daytime sleepiness emphasizes the potential impact of poor sleep quality on daytime functioning. These findings highlight the need for interventions targeting specific sleep-related behaviors among Nigerian medical students to improve sleep quality and overall well-being.^[29]^

Among the study population, academic level negatively correlated with sleep quality, suggesting that sleep quality declines as students progress to higher academic levels. This finding is consistent with previous studies highlighting the increased academic demands and stressors experienced by students as they advance in their medical education.^[30]^ The negative correlation between academic level and sleep quality emphasizes the need for targeted interventions to address sleep disturbances and promote better sleep among medical students at different stages of their education. Additionally, work during the day positively correlated with sleep quality, indicating that participants who engage in daytime work experience better sleep quality. On the other hand, work at night negatively correlates with sleep quality, underscoring the detrimental effects of night shift work on sleep quality and circadian rhythm disruption. Addressing the challenges associated with night shift work and implementing strategies to mitigate its impact on sleep quality are crucial considerations for medical education programs. Factors such as sleep latency, coffee consumption late at night, and the use of sleeping pills also showed correlations with sleep quality, suggesting the need for further research and exploration of these associations in the context of Nigerian medical students.

## 5. Limitations and strengths

This study has limitations that should be considered when interpreting the results. The cross-sectional design employed in this study limits the ability to establish causal relationships or determine the temporal sequence of events between sleep patterns and quality among Nigerian medical students. Longitudinal studies would provide more robust evidence on the directionality and potential changes in sleep patterns over time. Second, reliance on self-reported data introduces the possibility of recall and social desirability biases. Participants may have underreported or overreported certain sleep patterns or behaviors due to memory limitations or the desire to present themselves in a favorable light. Objective measures, such as polysomnography or actigraphy, could enhance the accuracy and reliability of the sleep data collected. Despite the limitations, this study has several notable strengths contributing to understanding sleep patterns and quality among Nigerian medical students. Firstly, the study addresses a significant gap in the literature by focusing specifically on this understudied population. Medical students in Nigeria face unique challenges and stressors, and examining their sleep patterns is essential for identifying potential areas of intervention and support. By highlighting the prevalence and potential impact of suboptimal sleep quality, the study raises awareness of the importance of sleep hygiene and the need for interventions to improve sleep among medical students in Nigeria. This knowledge can inform educational institutions and healthcare professionals in developing strategies to support medical students’ well-being and academic success.

## 6. Conclusion

Our study provides valuable insights into Nigerian medical students’ sleep patterns, behaviors, and quality. The findings highlight the challenges these students face in attaining adequate sleep and the potential impact on their cognitive function and overall well-being. Tailoring interventions to address specific factors such as gender, age, caffeine consumption, and work schedules is crucial for promoting optimal sleep quality and supporting Nigerian medical students’ academic success and well-being. Future research should continue to explore the underlying determinants of sleep quality, conduct longitudinal studies to understand the trajectory of sleep patterns throughout medical education and evaluate the effectiveness of interventions targeting modifiable factors. By improving sleep outcomes, medical education programs can contribute to Nigerian medical students’ overall health and success.

## Author contributions

**Conceptualization:** Nicholas Aderinto.

**Writing – original draft:** Nicholas Aderinto, Gbolahan Olatunji, Samson Afolabi, Abdulbasit Muili, Peter Olaniyi, Mariam Edun, Damilare Olakanmi.

**Writing – review & editing:** Nicholas Aderinto, Gbolahan Olatunji, Samson Afolabi, Abdulbasit Muili, Peter Olaniyi, Mariam Edun, Damilare Olakanmi.
